# Endometriosis-Associated Angiogenesis and Anti-angiogenic Therapy for Endometriosis

**DOI:** 10.3389/fgwh.2022.856316

**Published:** 2022-04-05

**Authors:** Monica S. Chung, Sang Jun Han

**Affiliations:** ^1^Division of Reproductive Endocrinology and Infertility, Department of Ob/Gyn, Baylor College of Medicine, Houston, TX, United States; ^2^Laboratory of Dan L. Duncan Cancer Center and Reproductive Medicine, Department of Molecular and Cellular Biology, Baylor College of Medicine, Houston, TX, United States

**Keywords:** endometriosis, angiogenesis, estrogen receptors, vascular endothelial growth factor, anti-angiogenic therapy

## Abstract

Endometriosis is a known estrogen-dependent inflammatory disease affecting reproductive-aged women. Common symptoms include pelvic pain, dysmenorrhea, dyspareunia, heavy menstrual bleeding, and infertility. The exact etiology of endometriosis is largely unknown, and, thus, the diagnosis and treatment of endometriosis are challenging. A complex interplay of many molecular mechanisms is thought to aid in the progression of endometriosis, most notably angiogenesis. This mini-review examines our current knowledge of the molecular etiology of endometriosis-associated angiogenesis and discusses anti-angiogenic therapy, in the blockade of endometriosis-associated angiogenesis, as potential non-hormonal therapy for the treatment of endometriosis.

## Introduction

Endometriosis is an estrogen-dependent inflammatory disease (1), defined as the presence of endometrial glands and stroma outside the uterine cavity. Endometriosis affects up to 5–10% of reproductive-aged women (2, 3) and has a higher prevalence in infertile women (4). The prevalence, however, may be underestimated because of diagnostic difficulty (3) and variation in clinical presentation. Early age at menarche, short menstrual cycle length, lean body size, and decreased parity are characteristically associated with a greater risk of endometriosis (3). Common symptoms that are predictive of the diagnosis of endometriosis include abdominopelvic pain, severe dysmenorrhea, dyspareunia, heavy menstrual bleeding, infertility, and a prior diagnosis of irritable bowel syndrome or pelvic inflammatory disease (5, 6). Clinicians should also suspect endometriosis in women of reproductive age with cyclical dyschezia, dysuria, or hematuria (6).

Diagnosis and treatment of endometriosis are challenging, given the large knowledge gap in specific cellular and molecular pathways. Surgical visualization and sampling with histologic review are necessary to confirm the diagnosis of endometriosis, which makes diagnosis more difficult. In addition, clinical presentation, treatment response, or prognosis do not frequently correlate with classification and staging (7, 8).

The exact etiology of endometriosis is largely unknown, but a few theories have been proposed and include retrograde menstruation, coelomic metaplasia, and lymphatic and vascular metastasis (9). Retrograde menstruation is a widely accepted proposed mechanism that refers to the overflow of menstrual debris, containing endometrial tissue, through the fallopian tubes and into the pelvic peritoneal cavity (10). Additional factors are necessary to explain retrograde menstruation however, since retrograde menstruation occurs in most reproductive-aged women, but only 10% of reproductive-aged women have a diagnosis of endometriosis. It is suggested that endometrial stromal cells provide adhesive ability through integrin and localized inflammatory responses compared to normal endometrial stromal cells (9).

Current medical treatment focuses on hormonal manipulation to induce a hypoestrogenic state in women. Common medications include combined oral contraceptive pills, progestins, and gonadotropin-releasing hormone (GnRH) agonists and antagonists (11, 12). However, their ineffectiveness, side effects, and recurrence after discontinuation often limit these options. In addition, adverse side effects, including hot flashes, memory loss, and insomnia are often associated with GnRH agonists (13). Surgical management for excision of endometriosis is an option for patients who desire relief of symptoms, but recurrence is commonly encountered postoperatively. Overall, safe, effective, non-hormonal targeted therapies are limited/inadequate for patients with endometriosis. This article aims to review our current knowledge of endometriosis-associated angiogenesis and discuss anti-angiogenic treatment as non-hormonal therapy for the treatment of endometriosis (14).

## Angiogenesis and Endometriosis

The precise pathways in the pathogenesis of endometriosis are complex and largely unknown. Various theories have been proposed as potential sources of endometriotic lesions (9), but the maintenance and progression of endometriosis entail a complex interplay of many molecular mechanisms. Immune dysregulation with localized inflammation, hyperproliferation, anti-apoptosis, and enhanced angiogenesis have a critical role in the progression of this disease. For instance, in endometriotic endometrial cells, TNFα-induced apoptosis signaling is effectively suppressed by steroid receptor coactivator-1 (SRC-1) isoform/ERβ axis (15–17). ERβ also causes inflammasome-mediated hyperproliferation of endometriotic lesions for the progression of endometriosis (16).

Angiogenesis is a physiologic process that provides fundamental vasculature for the overall growth and repair of organisms' systemic and local tissue needs. Highly regulated angiogenesis is essential for normal reproduction and plays a crucial role in follicular maturation, development of a functional corpus luteum, and endometrial growth (18). Angiogenic dysregulation, or the excessive growth of new blood vessels, can contribute to the establishment and progression of many diseases. In this frame of reference, angiogenesis has a critical role in the pathogenesis of endometriosis, because the growth of new blood vessels from pre-existing vessels is necessary for the survival and progression of ectopic endometrial implants. Limited information describing the precise mechanism of endometriosis-associated angiogenesis is available, however. Neovascularization from a complex system of cytokines, growth factors, steroids, and eucasanoids in the peritoneal environment, is thought to aid in recruiting new capillaries for the progression of ectopic lesions (18). Furthermore, mobilization and recruitment of bone marrow-derived endothelial progenitor cells (EPCs) to areas of hypoxic tissue, or “vasculogenesis,” also provides *de novo* formation of microvessels in endometriosis (19, 20). Vascular endothelial growth factor (VEGF) and fibroblast growth factor-2 were found to stimulate the mobilization of EPCs from bone marrow (19). Moreover, the dynamics of hypoxia with endothelial injury, inflammation, and ERα expression aid in the employment of EPCs for the growth of endometriotic implants (21, 22).

## Factors for Endometriosis-Associated Angiogenesis

The distinct interplay between cytokines, growth factors, and angiogenic factors aid in establishing and progressing endometriotic implants.

### Cytokine

The immune system, particularly Interleukin (IL)-1β, the dominant interleukin-1 secreted by activated peritoneal macrophages, stimulates stromal cells to produce angiogenic molecules (14). Interleukin (IL)-6, produced by endometriotic stromal cells in the presence of (IL)-1β, also increases angiogenic factors in neutrophils to stimulate endometriosis-associated angiogenesis (17, 23). IL-8, a pro-angiogenic factor, may potentiate neovascularization of ectopic implants, as elevation of IL-8 is observed in endometriosis patients (24, 25). IL-17A, in human endometriotic lesions, significantly increases angiogenic (VEGF, IL-8, IL-6, and IL-1β) and chemotactic cytokines (G-CSF, CXCL12, CXCL1, and CX3CL1) in endometrial cells (26). VEGF is a very potent and highly responsive angiogenic factor. Numerous factors aid in VEGF modulation, including Activin A (27) and IL-1β (14). VEGF protein expression is present in normal endometrial stromal cells, with levels increasing in response to estrogen and progesterone (17, 28). Cyclic VEGF expression is observed throughout the menstrual cycle and has the most significant expression during the secretory phase (28). Compared to women without endometriosis, increased VEGF levels are found in both peritoneal fluid of women with endometriosis and ectopic endometriotic tissue and contribute to the angiogenic microenvironment in endometriosis (29–31). In addition to endometriotic lesions, the VEGFR1/VEGF signaling in macrophages and fibroblasts enhance the growth of endometriotic lesions by activating lymphangiogenesis (32).

### Transcription Factors

Hypoxia-inducible factor enhances expression of pro-angiogenic factors, such as VEGF, in vascular endothelial cells to enhance hypoxia-induced angiogenesis (33, 34). Ovarian endometriomas have a higher level of HIF-1α compared to normal endometrium (35). In the presence of HIF-1α, VEGF mRNA expression levels increase in response to hypoxia; moreover, HIF-1α is required for oxygen-regulated transcriptional activation of genes encoding VEGF to enhance hypoxia-induced angiogenesis (36, 37). Thus, the HIF-1α/VEGF axis is critical in endometriosis-associated angiogenesis. In addition to angiogenesis, HIF-1α also promotes endometriotic stroma cell migration and invasion by up-regulating autophagy in endometriosis (38).

Since endometriosis is an estrogen-dependent disease, estrogen and estrogen receptors (ERs) are critical for the progression of endometriosis (39). Additionally, endometriotic tissue has higher local estradiol concentrations than normal endometrium by increasing steroidogenic factor I (SF-I) and aromatase (2, 10). In a preclinical model of endometriosis, targeting ERs, chloroindazole (CLI) for ERα and oxabicycloheptene sulfonate (OBHS) for ERβ, effectively suppresses endometriosis progression by inhibiting ER-dependent inflammatory activity (40). Additionally, PHTPP, a selective ERβ antagonist, effectively suppresses endometriosis progression in mice with endometriosis (16).

Estrogen is a pro-angiogenic hormone, widely known for its effects of neovascularization and angiogenesis in the uterus and endometrium through proliferation and migration of endothelial cells and formation of new matrices around vessels. Estrogen-mediated angiogenesis, however, is also necessary in non-reproductive tissue for wound healing, reestablishment of blood supply to ischemic tissue, tumor growth, and repair of damaged organs (41–43). Furthermore, endothelial progenitor cells, which play an important role in angiogenesis, are hormonally regulated (44).

**Figure 1:**
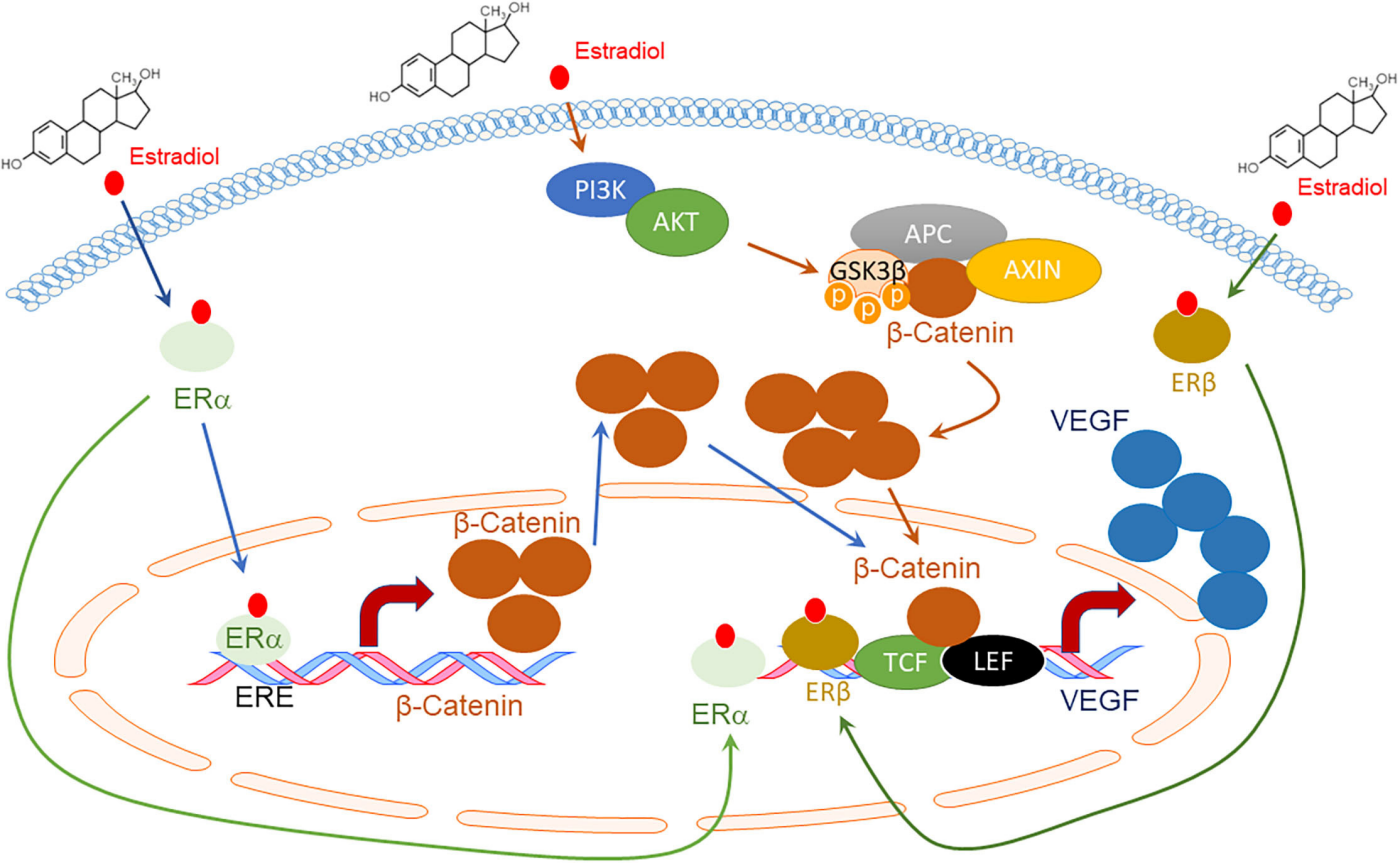
Schematic diagram of VEGF expression by Estradiol (E2) and Estrogen Receptors (ERs) through the Wnt/β-catenin signaling pathway. E2 promotes the direct binding of ERα to the Estrogen Response Element (ERE) site of the β-catenin promotor, enhancing its expression (45). E2 also activates the Phosphoinositide 3-kinases (PI3Ks)/AKT serine-threonine protein kinase (AKT) axis, which inactivates Glycogen synthase kinase (GSK) 3β through phosphorylation. The inhibited β-catenin destruction complex, which consists of APC regulator of WNT signaling pathways (APC) and Axis Inhibition Protein *(*AXIN), decreases β-catenin degradation. Accumulated β-Catenin enters the nucleus to bind to transcription factor 3/lymphoid enhancing binding factor 1 (TCF3/LEF1), enhancing VEGF expression (45). ERα and ERβ also directly bind to the VEGF promoter region and increase VEGF expression upon E2 activation (46–48).

What is the correlation between estrogen and angiogenesis for endometriosis progression? The 17β-Estradiol (E2) up-regulates VEGF expression in human primary endometrial stromal cells by activating the Wnt/β-catenin axis through ERs and thus enhances their ability to establish a new blood supply to the human exfoliated endometrium (Figure 1) (45, 49). Estrogen and selective agonist for each subtype of ER, such as ERα agonist, 4,4′,4″-(4-propyl-(1H)-pyrazole-1,3,5-tryl) triphenol and ERβ agonist 2,3-bis(4-hydroxy-phenyl)-propionitrile [DPN]) regulates the axonal guidance molecules of the SLIT/ROBO signaling that have a critical role in neuroangiogenesis occurring in endometriosis lesions found on the peritoneal wall (50). Furthermore, ERβ directly regulates the expression of genes involved in hypoxia-induced angiogenesis, such as HIF1α, VEGF, and Angiotensin (Ang)1 in ectopic lesions of mice with endometriosis to stimulate endometriosis progression (51). Therefore, E2/ERs axis has a critical role in regulating genes involved in endometriosis-associated angiogenesis in endometriotic lesions to promote endometriosis progression. In addition to E2, exposure of bisphenol A elevates ERβ in mouse endometrium, promoting endometriosis progression by activating ERβ-regulated endometriosis cellular pathways involving angiogenesis (52). Besides ERs, G Protein-Coupled Estrogen Receptor (GPER) levels are significantly elevated in endometriotic lesions and endometriosis-associated macrophages by stress-related hormones and inflammation (53, 54). Therefore, rapid estrogen effects mediated by GPER also have a critical role in the hormonal regulation of endometriosis.

Peptide hormones regulate angiogenesis by stimulation or inhibition to promote or prevent the growth of target tissue. Proteolysis converts the original hormone to either pro- or anti-angiogenic peptides. For example, growth hormone, prolactin, and placental lactogen family are structurally and functionally related, all released from the anterior pituitary. These hormones are pro-angiogenic when released, but upon proteolysis, they display angiogenic inhibitory properties. Endothelin, gonadotropins, insulin-like growth factor I (IGF-I), parathyroid hormone, and thyroid-stimulating hormone exhibit pro-angiogenic properties, whereas angiotensin, somatostatin, and natriuretic peptides demonstrate angiogenic inhibitory properties (55). Endothelin and IGF-1 may also play a role in endometriosis-associated angiogenesis as their levels were found to be significantly higher in women with endometriosis compared to controls (56, 57).

Although the exact molecular etiology of thyroid hormone and pro-angiogenesis has yet to be discovered, thyroid hormone has been implicated in both physiologic and pathologic angiogenesis in experimental models. Thyroid hormone-induced cardiac hypertrophy and ischemia models have demonstrated sustained angiogenesis and coronary blood flow. The hormone may also induce the expression of transcription factors that play a role in coronary artery collateralization in hypoxia (58). Furthermore, larger endometriotic implants were found with increased thyroid hormone levels, and increased chronic pelvic pain and disease score were noted in endometriotic patients with thyroid disorder (59). Given these findings, thyroid hormone may contribute to endometriosis-associated angiogenesis.

## Angiogenesis in Tumor Progression

Tumor angiogenesis differs significantly from physiologic angiogenesis. However, distinctions between angiogenesis in tumors vs. endometriosis are mainly unknown. Therefore, understanding tumor angiogenesis is essential for developing and advancing anti-angiogenic therapy, which has been employed as a potential treatment option for endometriosis.

Angiogenesis is constitutively activated during tumor progression to promote cancer cell progression by activating neovascularization (60). Physiologic angiogenesis is more tightly regulated and stabilizes once new vessels are formed. However, angiogenic tumor vessels are dilated and tortuous, and vascular density and blood vessel diameter are not uniform (61). Moreover, the tumor microenvironment favors hypoxic conditions. Therefore, the cancer-associated hypoxic conditions up-regulate Hypoxia-Inducible Factor (HIF)-1α transcription factor to increase its target genes, which include vascular endothelial growth factor (VEGF), platelet-derived growth factor (PDGF), placental growth factor (PlGF), and hepatocyte growth factor (HFG) in tumors to generate angiogenic tumor vessels (62–65).

The hypoxia /HIF1-α axis is required for normal endometrial repair during menstruation (66). In addition to normal endometrial function, endometriosis is associated with local angiogenic and hypoxic mechanisms, similar to cancer angiogenesis. For example, ovarian endometriomas express high levels of HIF-1/2α, and VEGF-A expression compared to endometrium of women without endometriosis (35). HIF-1α levels are elevated in ectopic endometrial lesions, and hypoxia plays a critical role in the survival of retrograde reflux of endometrial fragments during angiogenesis in early implanted ectopic endometrial lesions (67). Therefore, HIF1-α-mediated angiogenesis has a critical role in endometriosis-like cancer progression.

## Non-Angiogenic Pathways in Tumor Progression

Anti-angiogenesis therapy has demonstrated only modest success in cancer patients, and, thus, further efforts have been geared toward examining non-angiogenic modalities for tumor growth. Formation of blood vessels in cancer cells through non-angiogenic modalities that rely on alternative vascularization methods include Vascular mimicry, Vascular co-option of vessels, and Intussusceptive microvascular growth, or IMG (68).

Vasculogenic mimicry creates microvascular channels in tumor cells without the presence of endometrial cells, providing a network of fluid-conducting channels (69).

Vascular co-option of vessels is a non-angiogenic means for cancer cells to obtain a blood supply by hijacking pre-existing blood vessels in the surrounding tissue to support tumor growth and metastasis (70).

Vessel intussusception, or intussusceptive microvascular growth (IMG), generates new vascular structures by extending the capillary wall into the lumen of pre-existing vessels. Compared to angiogenesis, IMG is typically rapid as it doesn't rely on the proliferation of endothelial cells but rather on the remodeling of existing vascular structures (71). However, it has not been reported whether non-angiogenic pathways are also involved in endometriosis-associated angiogenesis.

## Anti-Angiogenic Therapy for Endometriosis Treatment

Angiogenesis has a critical role in endometriosis progression. Therefore, various angiogenic blockers have been employed as non-hormonal therapy for endometriosis (Table 1). For example, VEGF blockers and inhibitors have demonstrated promising results in mice, decreasing the number of endometriotic implants, reducing vascular density, increasing apoptosis, and reducing VEGF levels in peritoneal fluid (81, 121, 122). Similar results were achieved in a rat model without compromising ovarian reserve (120). Furthermore, in a human clinical trial, a patient was treated for severe endometriosis with Bevacizumab (Avastin, a monoclonal antibody directed against VEGF) and reported complete disappearance of her therapy-refractory dysmenorrhea. Diffuse fibrosis of her endometriosis lesions was also observed at second-look laparoscopy (123).

**Table 1:** Anti-angiogenic therapy and their mechanisms of action.

**Class of drugs**	**Name of drug**	**Anti-angiogenic effects on endometriosis**	**Mechanism of action**
Endogenous angiogenesis inhibitors	Angiostatin	Inhibits the number of endometriosis lesions (72)	Binding to ATP synthase, angiomotin, integrin, annexin II, angiostatin binding sequence protein, c-met and NG2 proteoglycan on the cell surface Binding to tissue plasminogen activator Inhibition of endothelial cell proliferation Induction of endothelial cell apoptosis Inhibition of VEGF and bFGF signaling (73, 74)
	Endostatin	Reduction of microvessel density Disruption of immature microvessels Inhibits the number of endometriosis lesions (75, 76)	Binding to Integrin and E-selectin Inhibition of endothelial cell proliferation and migraine Induction of endothelial cell apoptosis Blockade of VEGF signaling Pleiotropic action on many genetic pathways regulating angiogenesis (77–79)
Growth factor inhibitors	Anti-VEGF antibody	Reduction of microvessel density Disruption of immature microvessels (75, 76)	Neutralization of active VEGF and inhibits its activity (80)
	Bevacizumab	Reduction of VEGF levels in peritoneal fluid (81)	Recombinant humanized monoclonal antibody that inhibits VEGF (82)
	VEGF-targeted gene therapy	Induction of apoptotic cell death (83)	
	Soluble truncated VEGF receptors (Flt-1)	Disruption of immature microvessels Inhibits the growth of human endometrium in mice (84)	Neutralization of active VEGF and inhibits its activity (84)
	2-Methoxyestradiol	VEGF inhibitor Suppression of HIF-1α and VEGF expression (85)	Inhibition of the expression and transcriptional activity of HIF-Iα Induction of apoptosis and tubulin polymerization Induction of endothelial nitric oxide synthase (86)
	SU5416, SU6668	Reduction of microvessels Inhibition of vessel maturity in endometriotic lesions (22)	Selective inhibition of tyrosine kinase activity (87, 88)
Statins	Simvastatin	Reduction of microvessel density Suppression of MCP-I expression (89, 90)	Blockade of HMG-CoA reductase Inhibition of endothelial cell proliferation Induction of apoptosis Downregulation of VEGF synthesis Suppression of MMP secretion (91)
	Atorvastatin	Reduction of VEGF levels in peritoneal fluids Reduction of VEGF, RAGE, EN-RAGE and COX-2 expression Inhibition of VEGF in endometriotic stromal cells (92, 93)	
	Lovastatin	Inhibition of vascular sprouting Inhibition of VEGF in endometriotic stromal cells (94)	
PPAR agonists	Fenofibrate	Reduction of VEGF levels in peritoneal fluid (95)	Binding to PPAR-γ Energy homeostasis, metabolism, inflammation, and angiogenesis (96)
	Rosiglitazone	Suppression of VEGF expression (97)	
	Pioglitazone	Reduction of microvessel density (98)	
Immunomodulators	Lipoxin A4	Reduced activity of MMP-9 Decreased mRNA levels of VEGF Reduces size of endometriosis lesions Downregulates inflammation-associated proteins (IL-6, VEGF, matrix metalloproteinase 9) (99)	Anti-inflammatory effects Inhibition of VEGF-stimulated angiogenesis (100, 101)
	Pentoxifylline	Decreased expression of VEGF-C and Flk-I (101)	Pleiotropic action on the production of inflammatory mediators and the responsiveness of immunocompetent cells to inflamatory stimuli Suppression of VEGF signaling (100, 101)
	Rapamycin	Reduction of microvessel density, VEGF expression, and endothelial cell proliferation (22)	Inhibition of mTOR Inhibition of VEGF signaling (102)
Progestins, Danazol and GnRH agonists	Leuprolide acetate	Reduction of macrophage infiltration and microvessel density Increase in apoptotic cell death (103)	Binding to the GnRH receptor (104)
	Progesterone	Reduced proliferation of endometrial stromal cells Suppression of bFGF, VEGF-A, Cyr-61, and MMP expression (105)	Binding to steroid hormone receptors (105, 106)
	Danazole	Reduction of VEGF serum levels (107)	Induction of anovulation Increasing free testosterone (108)
Dopamine agonists	Cabergoline	Reduction of microvessel density and angiogenic gene expression Inhibition of VEGF and VEGFR-2 expression Suppression of VEGF and Notch-4 Up-regulation of Ang-I and Wnt (109–111)	Binding to dopamine D2 receptor Inhibition of VEGFR-2 phosphorylation Up-regulation of Ang-I and Wnt (109, 112)
	Quinagolide	Reduction of microvessel density and angiogenic gene expression Downregulation of VEGF/VEGFR2, CCL2, RUNXI, AGGFI, and PAI-I (110, 113)	Binding to dopamine D2 receptor Downregulation of VEGF/VEGFR-2, pro-angiogenic cytokines and PAI-I (113)
COX-2 inhibitors	Celecoxib	Reduction of vascularized lesion area (114)	Inhibition of COX-2, carbonic anhydrase, PDK I Induction of apoptosis (115)
	Rofecoxib	Reduction of VEGF levels in peritoneal fluid (116)	
	Parecoxib	Reduction of lesion size, microvessel density, number of macrophages Decreased expression of VEGF and Flk-I (117)	
Other	Macrophage migration inhibitory factor (MIF) antagonist	Reduces the expression of VEGF, cell adhesions receptors, MMP-2, MMP-9, IL-8, COX2 (118)	Inhibits cell adhesions, tissue remodeling, angiogenesis, and inflammation (118)
	Retinoic acid	Decreases the volume of endometriotic implants (119, 120)	Direct downregulation of VEGF production (120)

Other assuring anti-angiogenic therapies have been investigated. For example, macrophage migration inhibitory factor (MIF) demonstrates the development of endometriosis *in vivo* and demonstrated pro-apoptosis activity (118, 121). Retinoic acid has known anti-angiogenic characteristics, suppressing the growth of endometriotic lesions and inhibiting peritoneal cytokine secretion in an immunocompetent mouse model (119, 121). Statins inhibit inflammation and angiogenic genes, cyclooxygenase-2 and VEGF, in endometriotic stromal cells (92, 121). Cabergoline, a dopamine agonist, inhibits the development of endometriosis by inhibiting VEGF and VEGFR-2 (109, 121) and may prove an effective therapy for women with chronic pain due to endometriosis (124).

Furthermore, women with endometriosis display significantly elevated levels of ERβ in ovarian endometrioma when compared to normal endometrium (125). ERβ overexpression could potentiate infertility in women with endometriosis because ERβ overexpression impairs decidualization in the stroma of their endometrium in mice (16). ERβ also has a critical role in the progression of endometriotic lesions, including angiogenesis (51). Therefore, targeting ERβ could benefit both regression of ectopic implants by inhibiting proliferation and angiogenesis of endometriotic lesions and optimizing endometrial receptivity in patients with endometriosis. The 2-methoxyestradiol is a natural metabolite of estradiol and binds to GPER, but not ERs (126). The 2-methoxyestradiol suppresses the growth of ectopic lesions in mice model of endometriosis (85) by inhibiting angiogenesis of endometriosis progression because 2-methoxyestradiol downregulates angiotensin AT1 receptor (127). Also, 2-methoxyestradiol suppressed HIF-1α expression *in vivo*, results in a decreased expression of HIF-1α target genes, such as VEGF, phosphoglycerate kinase, and glucose transporter-1 (85). Therefore, targeting GPER can be employed as anti-angiogenic therapy for endometriosis treatment.

## Modes of Resistance to Anti-Angiogenic Therapies

Although angiogenesis is a critical regulator in the progression of malignant tumors, individuals may respond differently to anti-angiogenic therapy. These anti-angiogenic agents are not equally active across all tumor types. Few clinical studies support the theory that anti-angiogenic treatments can prevent activation of the angiogenic switch in the progression and metastasis of tumors (128). Furthermore, anti-angiogenic biomarkers do not adequately represent a specific response to these therapeutic agents (129).

Anti-angiogenic therapy has resulted in transient improvements in cancer treatment with tumor stability or shrinkage and increased survival. Over time, however, the tumor re-establishes its growth and progression, challenging the notion that angiogenesis is necessary for the advancement of tumors. Adaptive, or evasive, resistance and intrinsic, or pre-existing, non-responsiveness have been suggested as distinct pathways in evading anti-angiogenic therapy. Adaptive resistance theorizes that angiogenic tumors can adapt to the presence of an anti-angiogenic agent and obtain mechanisms to escape the inhibition of angiogenesis. Comparably, intrinsic resistance likely encompasses a similar mechanism, but the tumor may have underlying characteristics (type of tumor, stage of progression, treatment history, individual genotype) that place an individual at greater risk. In addition, those with pre-existing non-responsiveness typically see no clinical benefit with angiogenesis inhibitors, whereas those with evasive non-responsiveness demonstrate transient benefit (130).

Although resistance to anti-angiogenic therapy has also been noted, interestingly, in some cases, acquired resistance may be a transient occurrence. Sequential therapy with a similar, but not identical, anti-angiogenic drug is a strategy to delay the onset of acquired resistance or treat cancers that have progressed through anti-angiogenic therapy (131).

## Discussion

Non-hormonal targeted therapy for endometriosis is critical for caring for women with endometriosis to circumvent the adverse effects of current hormonal treatment. Our present knowledge of endometriosis-associated angiogenesis suggests that anti-angiogenesis therapy can aid in targeted treatment for these patients. The proposed pathogenic pathways discussed in this article may assist in developing targeted therapies through adequately powered studies and a multidisciplinary approach.

Anti-angiogenic therapy may adversely impact normal physiologic angiogenesis, such as ovulation and wound healing (132), leading to adverse effects on reproductive function and teratogenicity during the treatment of endometriosis in reproductive-aged women. Thus, it is crucial to define the distinct angiogenic pathways specific for endometriosis to mitigate these potential side effects. ERβ and GPER are highly elevated in endometriotic tissue, compared to normal tissue, and could serve as new molecular therapeutic targets to suppress endometriosis-specific angiogenesis.

Smaller anti-angiogenic molecules, derived from natural products, have significant advantages over synthetic inhibitors. For instance, polyphenols, polysaccharides, alkaloids, terpenoids, and saponins containing natural products target tumor angiogenesis (133). Furthermore, smaller anti-angiogenic natural products can be more easily manufactured, confer lower cost, provide higher efficacy, and have little to no known toxicity (134). Ultimately, it is imperative to conduct further studies to explore natural angiogenesis inhibitors and evaluate its anti-angiogenic efficacy as a non-hormonal therapy for endometriosis treatment in the future.

## Author Contributions

MC drafted the manuscript. SH revised the manuscript. Both authors contributed to the article and approved the submitted version.

## Funding

This work was supported by grant funding from NICHD (5R01HD098059).

## Author Disclaimer

This manuscript review does not necessarily reflect the opinions or views of Baylor College of Medicine.

## Conflict of Interest

The authors declare that the research was conducted in the absence of any commercial or financial relationships that could be construed as a potential conflict of interest.

## Publisher's Note

All claims expressed in this article are solely those of the authors and do not necessarily represent those of their affiliated organizations, or those of the publisher, the editors and the reviewers. Any product that may be evaluated in this article, or claim that may be made by its manufacturer, is not guaranteed or endorsed by the publisher.
